# Hemorrhagic Shock Induces a Rapid Transcriptomic Shift of the Immune Balance in Leukocytes after Experimental Multiple Injury

**DOI:** 10.1155/2021/6654318

**Published:** 2021-01-27

**Authors:** Lisa Debler, Annette Palmer, Sonja Braumüller, Bettina Klohs, Tom Eirik Mollnes, Karlheinz Holzmann, Markus Huber-Lang, Rebecca Halbgebauer

**Affiliations:** ^1^Institute of Clinical and Experimental Trauma Immunology, University Hospital Ulm, Ulm, Germany; ^2^Department of Immunology, Oslo University Hospital, and University of Oslo, Norway; ^3^Center of Molecular Inflammation Research, Norwegian University of Science and Technology, Trondheim, Norway; ^4^Research Laboratory, Nordland Hospital, Bodø, and K.G. Jebsen TREC, University of Tromsø, Tromsø, Norway; ^5^Core Facility Genomics, Ulm University, Ulm, Germany

## Abstract

The immune response following trauma represents a major driving force of organ dysfunction and poor outcome. Therefore, we investigated the influence of an additional hemorrhagic shock (HS) on the early posttraumatic immune dysbalance in the whole population of blood leukocytes. A well-established murine polytrauma (PT) model with or without an additional pressure-controlled HS (mean arterial pressure of 30 mmHg (±5 mmHg) for 60 mins, afterwards fluid resuscitation with balanced electrolyte solution four times the volume of blood drawn) was used. C57BL/6 mice were randomized into a control, PT, and PT + HS group with three animals in each group. Four hours after trauma, corresponding to three hours after induction of hemorrhage, RNA was isolated from all peripheral blood leukocytes, and a microarray analysis was performed. Enrichment analysis was conducted on selected genes strongly modulated by the HS. After additional HS in PT mice, the gene expression of pathways related to the innate immunity, such as IL-6 production, neutrophil chemotaxis, cell adhesion, and toll-like receptor signaling was upregulated, whereas pathways of the adaptive immune system, such as B- and T-cell activation as well as the MHC class II protein complex, were downregulated. These results demonstrate that an additional HS plays an important role in the immune dysregulation early after PT by shifting the balance to increased innate and reduced adaptive immune responses.

## 1. Introduction

Hemorrhagic shock (HS) is a form of hypovolemic shock caused by severe blood loss, resulting in oxygen demand surpassing oxygen supply at a cellular level. Hemorrhage is accountable for an estimated 1.9 million deaths per year worldwide, from which 1.5 million result from physical trauma [1]. As traumatic injury affects mostly the young with road injury being the leading cause of death worldwide in the age group from 5 to 29 years, HS in trauma patients results in nearly 75 million years of life lost each year [1, 2]. In patients after massive transfusion, in-hospital mortality is as high as 23%, and a considerable number of survivors remains unable to return to work until one year after trauma [3]. These numbers demonstrate the necessity of improving immunopathophysiological understanding and deduced early diagnosis and effective treatment of HS. Preclinical and initial clinical care represent the most efficient levers for improvement, as the median time until death in HS patients is two hours [4]. Patients surviving the first hours after injury still need to overcome the mid- and long-term impacts, as HS increases the incidence of complications in severely injured patients by leading to systemic inflammation and inducing end-organ damage, for example, resulting in acute respiratory distress syndrome or multiple organ failure [5, 6]. Oxygen deprivation and a breakdown of blood-organ barriers lead to organ damage, and an additional release of the body's own damage-associated molecular patterns (DAMPs) in response to tissue injury and hypoxia contributes to a systemic inflammatory response [6–9]. These endogenous alarmins, which are released from the necrotic tissue as well as activated immune cells, further stimulate the cellular innate immunity via binding to surface pattern recognition receptors. Neutrophils, as the most abundant leukocytes in human circulation, play a key role in the initial inflammatory response to trauma and hemorrhage and subsequent repair processes through mechanisms like phagocytosis, generation of extracellular traps, release of reactive oxygen species, and production of cytokines. However, alterations in neutrophil function, phenotypic markers, and life span have been observed following severe trauma, potentially contributing to trauma-associated complications [10, 11]. Concerning the posttraumatic immune response, Xiao et al. showed that after critical injury, the expression of pathways involved in activation of pro- and anti-inflammatory innate immune reactions was strongly induced in peripheral blood leukocytes early after the insult, while pathways responsible for adaptive immunity were widely downregulated, in what was described as a “genomic storm.” In this study, patients who suffered from complications showed a longer time until the genomic response returned to the baseline [12]. These new insights lead to a shift in paradigm from the understanding that trauma leads to a systemic inflammatory response syndrome (SIRS) temporally followed by a counteracting compensatory anti-inflammatory response to a new hypothesis proposing rather a rapid induction of innate pro- and anti-inflammatory immune responses with a simultaneous impairment of adaptive responses after injury. Similarly, Lederer et al. observed significant alterations in the gene expression in leukocytes during the time course following murine trauma/hemorrhage starting 2 h after injury, with changes present especially in genes related to cell death and impairments in the immune response [13]. In patients, major tissue injury due to extensive surgery induced differential gene expression in blood leukocytes with upregulation of pathways of innate immunity and inflammation while genes involved in adaptive immunity, especially antigen presentation and T-cell function, were significantly downregulated [14]. In line, a recent study used a transcriptomic approach of the blood leukocyte gene expression in patients after severe trauma to predict adverse outcome [15]. As a likely result of these alterations in the gene expression, different leukocyte subpopulations such as neutrophils, monocytes, and regulatory T-cells were described to be functionally impaired in the critically ill, resulting in an increased risk of secondary infection [16].

Even though the clinical relevance of immune dysregulation after polytrauma (PT) and HS is widely acknowledged and precise epidemiological data underline its importance, the exact molecular mechanisms of the shift in immune balance after PT and especially the effect of an additional HS still remain unclear. To address the pro- and anti-inflammatory immune response of both the innate and adaptive branches after trauma, and to investigate specifically the impact of an additional hemorrhagic shock, we used a transcriptomic approach in a highly standardized, clinically relevant PT + HS mouse model.

## 2. Materials and Methods

### 2.1. Murine PT Model with HS

We performed a randomized prospective study strictly following the experimental protocol described in Denk et al. [17]. All procedures were performed in accordance with the guidelines of the National Institute of Health on the care and use of animals, and the protocol was approved by the University Animal Care Committee and the Federal Authorities for animal research, Tuebingen, Germany (No. 1194). C57BL/6 mice purchased from Jackson Laboratories (Bar Harbour, USA) with a mean body weight of 25 g (±2.5 g) and an age of 8 to 9 weeks were randomized into a polytrauma (PT), polytrauma with hemorrhagic shock (PT + HS), and a healthy control group without manipulation (Ctrl) with three animals in each group. Anesthesia was induced with 2.5% sevoflurane and 97.5% oxygen and kept up until the end of the observation period. Buprenorphine was given to trauma groups by subcutaneous injection for analgesia. The polytrauma included a traumatic brain injury, a closed transverse femoral fracture with soft tissue injury, and a blunt bilateral chest trauma. A femoral artery catheter was inserted to allow controlled blood loss and monitor blood pressure; a jugular vein catheter was inserted for fluid resuscitation and administration of catecholamines. Hemorrhage was induced one hour after trauma through controlled blood loss for 5 to 10 mins until a mean arterial blood pressure (MAP) of 30 mmHg (±5 mmHg) was reached and then maintained at this level for 60 mins. Afterwards, fluid resuscitation with balanced electrolyte solution four times the volume of blood taken was performed over 30 mins. Anesthesia and norepinephrine were adjusted in a standardized manner during the two-hour observation period to keep the MAP above 50 mmHg. Cardiac puncture was performed four hours after the induction of trauma in PT groups and directly after the induction of anesthesia in control animals.

### 2.2. RNA Isolation and Microarray Analysis

Blood was collected in RNAprotect Animal Blood Tubes (Qiagen, Hilden, Germany). After isolation of RNA using the RNeasy Protect Animal Blood Kit (Qiagen), samples with an RNA integrity number ≥ 9.1 were used. 200 ng total RNA and 5.5 *μ*g single-stranded DNA were used per hybridization in a GeneChip Fluidics Station 450 (Affymetrix, Santa Clara, USA) for microarray analysis. Single-stranded DNA was hybridized to Mouse Gene 1.0 ST GeneChip Arrays and scanned using a GeneChip scanner 3000 (both Affymetrix) after RNA amplification and labeling. Analysis of images was performed using Affymetrix Expression Console Software and BRB-ArrayTools. Using the robust multiarray average, normalized values of the raw feature data and log2 intensity expression summary values were calculated.

### 2.3. Selection of Genes

Genes that were significantly differently regulated through the additional HS were selected. As genes with an EntrezID but without gene symbol were excluded, 645 genes were initially selected for the analysis. 334 of the 359 up- and 262 of the 288 downregulated genes had a uniquely mapped ID on Gene Ontology and were therefore included in the enrichment analysis. A Venn diagram demonstrating the number of genes differently regulated in the comparisons between the experimental groups was designed using BioVenn (http://www.biovenn.nl) [18].

### 2.4. Enrichment Analysis and Heat Maps

The enrichment analysis was conducted with Gene Ontology (http://geneontology.org) with a PANTHER overrepresentation test being performed. The annotation version PANTHER 14.1 released March 12, 2019 with the annotation data set “PANTHER GO slim biological process” was used to identify the overexpression of the pathway innate immune response in upregulated genes. The annotation version GO Ontology database with the release date December 9, 2019 and the annotation data set “GO biological process complete” was used for further evaluation of upregulated genes. For the analysis of downregulated genes, the same annotation versions were used. The annotation data set “PANTHER pathways” identified the overrepresentation of B- and T-cell activation, and the annotation data set “GO cellular component complete” determined the overrepresentation of the MHC class II protein complex in the list of downregulated genes. Analyzed genes were tested against the reference list of the microarray. Heat maps were designed using Genesis (Alexander Sturn and Rene Snajder, TU Graz, version 1.8.1) [19]. The gene expression was normalized, sorted by trend of the expression value, and displayed with a set upper maximum value of +2 and a lower maximum value of -2.

### 2.5. Statistics

*t*-test was used to determine differentially expressed probe sets, and statistical significance was defined as *p* < 0.05 and a 1.5-fold change in the class comparison between the experimental groups. A *p* value <0.05 was used as a cut-off value in the enrichment analysis. For statistical analysis of overrepresentation, Fisher's exact test with FDR correction was applied. Results are presented as mean ± standard error.

## 3. Results

### 3.1. Mimicking and Monitoring of PT and HS in the Mouse Setting

**Figure 1:**
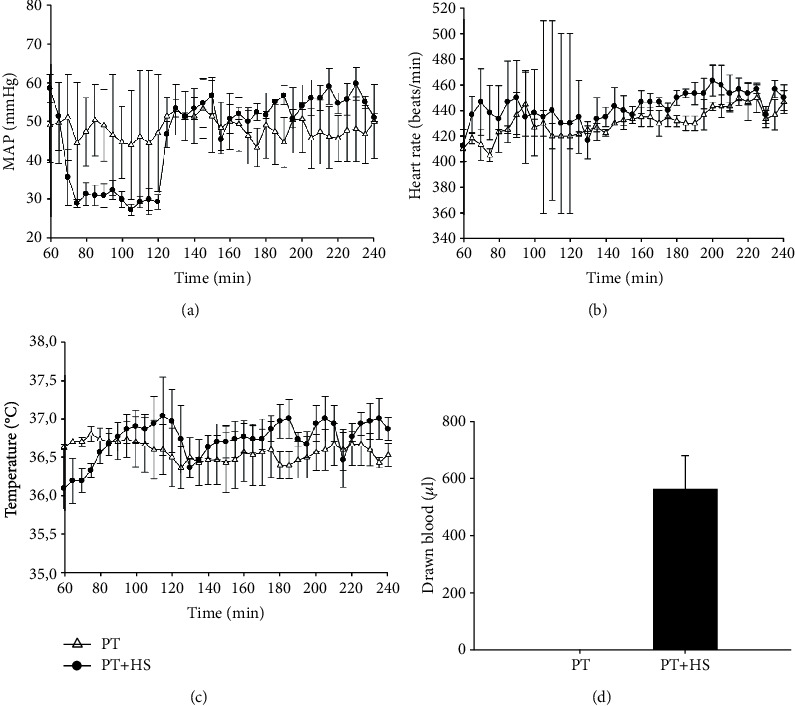
Mean arterial pressure (MAP) (a), heart rate (b), body temperature (c), and volume of blood drawn for the induction of the hemorrhagic shock (d) in mice after polytrauma (PT) and polytrauma with hemorrhagic shock (PT + HS).

To monitor hemodynamic changes, heart rate and MAP as well as body temperature were measured every 5 mins starting after the instrumentation of the mice (Figure 1). The MAP in PT mice remained stable at around 48 mmHg, whereas in PT + HS mice, the MAP following the induction of the hemorrhage was kept at a median of 30 mmHg for 60 mins and afterwards raised to and maintained at a median of 53 mmHg according to the study protocol (Figure 1(a)). PT + HS mice required an average 0.15 *μ*g (±0.07 *μ*g) of norepinephrine to reach or maintain the blood pressure target of 50 mmHg or greater. The heart rate of both the PT and PT + HS mice was rather stable (Figure 1(b)). Body temperature was maintained around 37°C in both groups by close control and a feedback loop device (Figure 1(c)). For the induction of the pressure-controlled HS, an average of 560 *μ*l (±120 *μ*l) blood was drawn from each animal (Figure 1(d)).

### 3.2. Additional HS in PT Mice Leads to an Altered Gene Expression in Leukocytes Four Hours after Trauma

**Figure 2:**
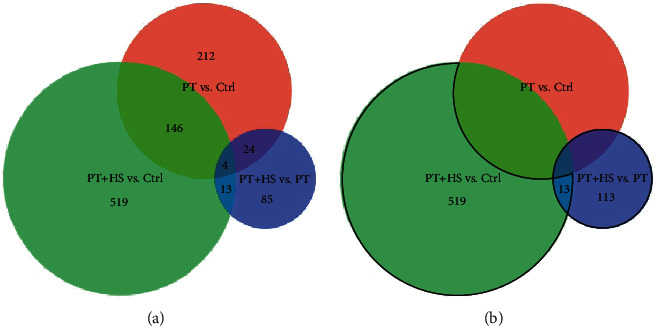
Venn diagram demonstrating the number of differentially expressed genes after exclusion of genes without gene symbols in the comparisons between the three experimental groups (a). Genes included in the enrichment analysis are bordered in black (b). Significantly up- and downregulated genes in the class comparison between the groups PT + HS and PT (blue, 126 genes) were analyzed. Genes up- or downregulated in the group PT + HS (green) but not in the group PT (red), both times compared to the Ctrl group, were also included (532 genes). 13 genes were duplicates as they fulfilled both the inclusion criteria.

The comparison between the groups PT and PT + HS showed a significantly different expression in 147 genes. PT animals compared to control animals exhibited a change in regulation of 466 genes and in a comparison between the groups PT + HS and Ctrl 904 genes were differently expressed. To specifically evaluate the influence of the additional hemorrhagic shock on the immune dysbalance after trauma, relevant genes were selected as shown in Figure 2. First, genes without an existing gene symbol were excluded; numbers of genes with gene symbols are shown in Figure 2(a). For further analysis, we selected 126 genes from the comparison between the two groups PT + HS and PT, with 77 up- and 49 downregulated genes in the PT + HS group compared to the PT group. To broaden our analysis, genes which were significantly differently regulated in the class comparison between the groups PT + HS and Ctrl but showed no significant difference between the groups PT and Ctrl were also included. As a result, 291 up- and 241 downregulated genes were added into the analysis. 13 of these genes were duplicates as they were already included in the comparison between PT + HS and PT animals. Using these criteria, the expression of 645 genes was significantly altered four hours after trauma through the additional HS (Figure 2(b)).

### 3.3. Upregulated Genes Relate to the Innate Immunity

**Figure 3:**
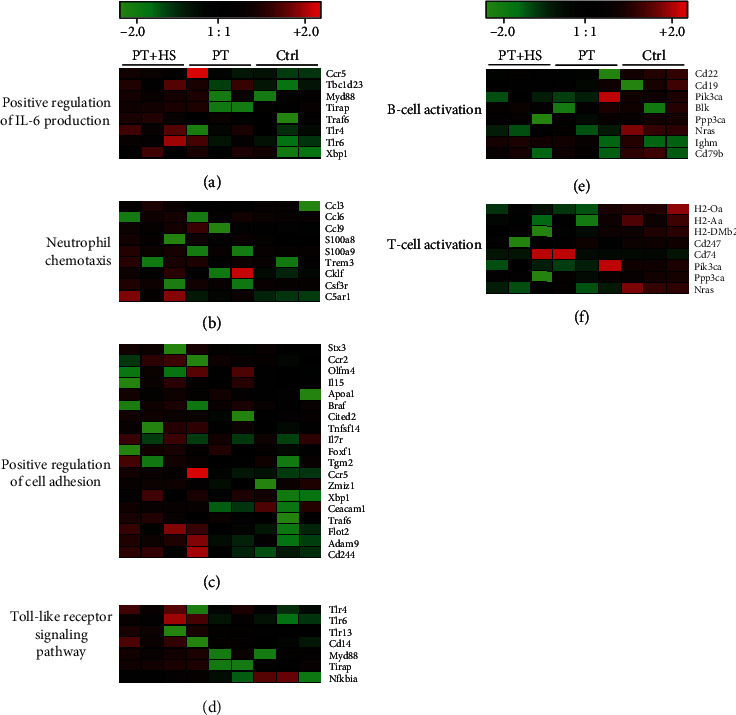
Heat map demonstrating the gene expression in the three experimental groups PT + HS, PT, and Ctrl; each column represents one animal. The high expression value of genes is shown in red and the low expression in green. Significantly upregulated genes of the pathways positive regulation of IL-6 production (a), neutrophil chemotaxis (b), positive regulation of cell adhesion (c), and toll-like receptor signaling pathway (d), as well as significantly downregulated genes of the pathways B-cell activation (e) and T-cell activation (f) are shown.

An enrichment analysis was performed with Gene Ontology on the 359 selected upregulated genes, from which 334 had a uniquely mapped ID and were further analyzed. Upregulated pathways strongly affected the innate immune response (fold enrichment (FE) 3.12, *p* = 9.28*E* − 08). Further evaluation showed a high expression of myeloid leukocyte activation (FE 8.14, *p* = 5.95*E* − 12) and differentiation (FE 5.67, *p* = 1.38*E* − 06). A positive regulation of cytokine secretion (FE 4.28, *p* = 4.75*E* − 05), specifically of IL-6 (FE 6.18, *p* = 4.26*E* − 06) (Figure 3(a)), IL-1*β* (FE 7.71, *p* = 2.32*E* − 04), and tumor necrosis factor (TNF) production (FE 5.37, *p* = 3.53*E* − 05), as well as an enhanced expression of the cytokine-mediated signaling pathway (FE 4.12, *p* = 5.16*E* − 07), was found. Upregulated genes involved in the positive regulation of IL-6 production included amongst others the chemokine receptor CCR5, X-box binding protein 1 (XBP1), and toll-like receptors (TLR) 4 and 6. Genes involved in neutrophil chemotaxis (FE 7.21, *p* = 1.05*E* − 05) (Figure 3(b)), such as the chemokines CCL-3, -6, and -9, the S100 proteins A8 and A9, and the complement receptor C5aR1, as well as in the positive regulation of cell adhesion (FE 2.77, *p* = 1.92*E* − 05) (Figure 3(c)), including the chemokine receptors CCR-2 and -5 and ADAM9 (a disintegrin and metalloproteinase domain-containing protein 9), were also highly expressed. Upregulated pathways further included the toll-like receptor signaling pathway (FE 7.12, *p* = 1.09*E* − 04) with an overexpression of TLR 4, 6, and 13, as well as CD14, myeloid differentiation primary response gene 88 (MYD88), toll-interleukin 1 receptor domain containing adaptor protein (TIRAP), and NF*κ*B inhibitor alpha (NFKBIA) (Figure 3(d)). Regarding intracellular signaling, a positive regulation of the JNK cascade (FE 4.47, *p* = 3.17*E* − 05), the ERK1 and ERK2 cascades (FE 3.21, *p* = 3.49*E* − 04), the stress-activated MAPK cascade (FE 4.04, *p* = 3.99*E* − 05), and the I*κ*B kinase/NF*κ*B signaling (FE 4.23, *p* = 4.54*E* − 04) was found.

### 3.4. Downregulated Genes Affect Pathways of the Adaptive Immune System

**Figure 4:**
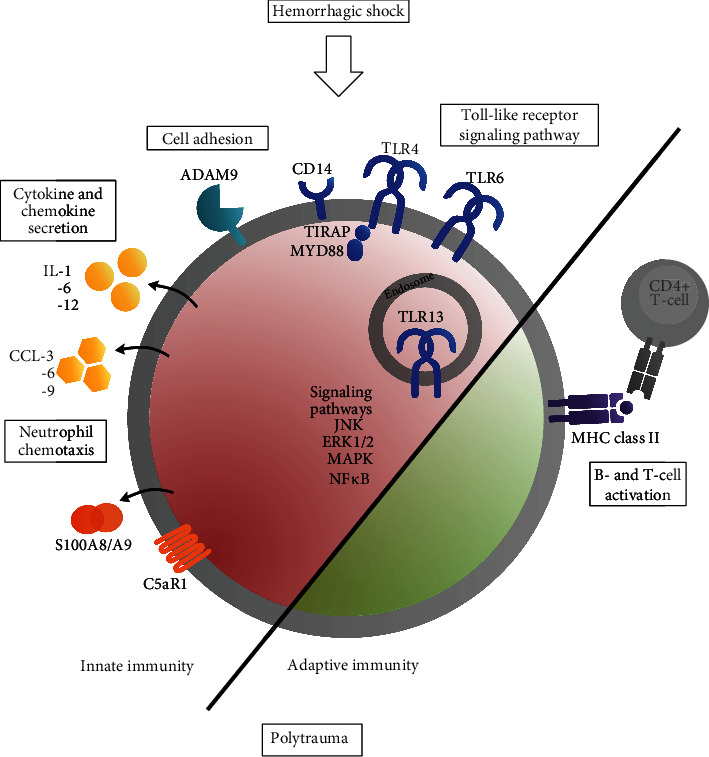
Selected pathways up- and downregulated by the additional hemorrhagic shock. Upregulated pathways (red) include cytokine and chemokine secretion, neutrophil chemotaxis, cell adhesion, and the toll-like receptor signaling pathway and affect mainly the innate immune response. Signaling pathways such as the JNK, ERK1/2 and MAPK cascades, and NF*κ*B signaling were also upregulated. Downregulated pathways (green) affect largely the adaptive immunity, such as B- and T-cell activation as well as the MHC class II protein complex.

Evaluation of the 288 downregulated genes, from which 262 had a uniquely mapped ID and were included in the enrichment analysis, showed that significantly modulated pathways relate largely to the adaptive immune system. Pathway analysis showed a downregulation of B-cell (FE 7.31, *p* = 2.68*E* − 05) (Figure 3(e)) and T-cell activation (FE 6.12, *p* = 8.53*E* − 05) (Figure 3(f)), as well as of the MHC class II protein complex (FE 33.99, *p* = 1.84*E* − 06). Downregulated genes included different B-cell receptors or antigens, such as CD19, CD22, CD79b (B-cell activation) and HLA class II histocompatibility antigens playing a role in MHC class II protein complex binding, such as H2-Aa, H2-Oa, and H2-DMb2 (T-cell activation). CD74 (fold change PT + HS vs. Ctrl: 0.44), also known as HLA class II histocompatibility antigen gamma chain, and MS4A1 (Fold Change PT + HS vs. Ctrl: 0.25), also known as CD20, a B-cell surface molecule, were also downregulated. Selected significantly up- and downregulated pathways are shown in summary in Figure 4.

## 4. Discussion

As molecular phenotyping technologies became more and more available, the idea of a gene panel being used in the clinical routine of trauma care has evolved [20, 21]. Besides potential new diagnostic markers, novel therapeutic targets can also be investigated through gene expression analysis of patients and in experimental animal models. In the present study, we took advantage of these techniques and showed, using the whole blood leukocyte population, that innate immunity genes were upregulated, and adaptive genes were downregulated following trauma with additional hemorrhagic shock.

Our PT mouse model with HS has been specifically developed to mimic in a highly standardized manner a hemodynamically stable or instable polytrauma. The combined injury affected the head, chest, and lower extremities with an Abbreviated Injury Scale (AIS) of 3, 3-4, and 3, respectively, and therefore resulted in an estimated Injury Severity Score (ISS) of 27 to 34 [17]; therefore, our model represents a trauma cohort with a rather severe injury pattern.

A “genomic storm” with an altered expression of over 80% of the leukocyte transcriptome over the first 28 days has been described in patients after severe blunt trauma, as well as burn injury and endotoxemia. Amongst the ten gene pathways with the highest increase in expression after injury were integrin signaling, leukocyte extravasation, toll-like receptor signaling, and IL-6 signaling [12]. Here, a parallel can be drawn to our experimental study, as the additional hemorrhage also lead to an overexpression of closely related pathways. In evaluated patients, upregulation of IL-6 and p38 MAPK signaling as well as downregulation of antigen presentation and T-cell regulation was associated amongst others with a complicated recovery. As these pathways were also highly expressed in our analysis, this could indicate a pivotal role of hemorrhage in increasing complications. However, clinical parameters associated with a poor recovery, such as high ISS, massive blood transfusion, or pronounced shock assessed by base deficit, had a surprisingly limited effect on the gene expression in these trauma patients, questioning hemorrhage as a main culprit of immune dysregulation after trauma [12].

Another study, focusing on the hyperacute time window after critical injury in patients, postulated that the early change in genomic activation is crucial for the later development of MODS. It was hypothesized that shock could act as a major driver of the early change in the genomic response, as patients who showed higher base deficits and lactate levels at admission demonstrated significantly altered gene expression in peripheral leukocytes immediately after injury and later developed MODS [22]. As there was a clear shift in the immune transcriptomic profile due to the hemorrhagic shock, our analysis confirms this hypothesis and clearly points to a significant role of hemorrhage in trauma-induced immune dysbalance. The impact of an additional hemorrhagic shock on the development of organ injury and dysfunction after trauma has also been recently demonstrated by our group [17].

Increased levels of IL-6 have been detected in trauma patients with hemorrhagic shock compared to severely injured patients without hemorrhage [6]. Likewise, the additional hemorrhagic shock in our experimental mouse model leads to an overexpression of genes involved in the positive regulation of IL-6 production. IL-6 acts as a keystone cytokine in the posttraumatic immune response with context-dependent pro- and anti-inflammatory effects; the assessment of serum IL-6 concentrations within the first 24 hours after trauma has shown potential in predicting posttraumatic complications, such as MODS and mortality [23].

Neutrophils are the first cells recruited to a site of inflammation or tissue damage as part of the innate immune response, guided by the release of chemoattractants, such as chemokines, complement anaphylatoxins, lipid derivates, and N-formylated peptides from different origins [24, 25]. The complement activation product C5a, a most potent chemotactic anaphylatoxin, interacts amongst others with the G-protein-coupled receptor C5aR1, which was upregulated in PT + HS animals. Blockage of the C5a-C5aR1 axis has been shown to result in improved neutrophil and organ function as well as outcome after experimental sepsis [26, 27]. C5a signaling through C5aR1 results in a strong proinflammatory response. A second C5a receptor has been described, C5aR2, which interestingly seems to counteract the strong C5aR1 response, and it remains to be shown what relative relevance these two C5a receptors have under pathophysiological conditions like trauma [28]. However, the translation of these results into the clinical context of trauma and HS is still pending. S100A8 and A9, both upregulated through the additional HS in our analysis, are released during activation of phagocytes and act mainly as the heterodimer calprotectin as endogenous activators of TLR4, as well as being involved in chemokine secretion and the recruitment of leukocytes [29–31]. Calprotectin is seen as a candidate diagnostic biomarker for inflammatory or autoimmune diseases; fecal calprotectin is already used in the clinical routine as a diagnostic marker for inflammatory bowel disease. In human sepsis and septic shock, S100A8/A9 was proposed as a diagnostic and prognostic marker, and the potential role as a therapeutic target is still a topic of ongoing research [32, 33].

Besides chemotaxis, cell adhesion plays a further crucial role in leukocyte recruitment, as both enable leukocyte extravasation and migration to the site of inflammation or tissue damage [34]. Overexpressed genes involved in cell adhesion included ADAM9, a member of the ADAM (a disintegrin and metalloprotease domain) family participating in cell-cell and cell-matrix interactions. Through its metalloprotease activity and interaction with integrins, ADAM9 modulates cell adhesion and promotes neutrophil chemotaxis and activation [35]. It has been proposed as a biomarker for tissue damage and shown to be of particular use in excluding a confounding effect of an infection, as in vitro exposure to pathogen-associated molecular patterns (PAMPs) leads to a very limited change in the expression of ADAM9, in contrast to many other biomarkers. [36].

In our model, hemorrhage leads to an upregulation of the toll-like receptors 4, 6, and 13 and further genes involved in the TLR signaling pathway. Toll-like receptors are genetically conserved pattern recognition receptors, and its ligands include different PAMPs and host-derived DAMPs. TLR4 mediates systemic inflammation and end-organ damage after trauma, but also plays a central component in noninfectious pathophysiological processes [37, 38]. Eritoran, an inhibitor of the TLR4 function, has been proposed as a promising treatment option for patients with trauma and hemorrhage to prevent multiple organ failures, as it suppressed HS-induced inflammation and organ damage in a mouse model of hemorrhage and reperfusion with reduced liver damage and gut barrier permeability, as well as an attenuated release of IL-6 [39–41]. However, in a phase 3 trial, Eritoran failed to reduce mortality in severe sepsis [42].

In the present study, we observed a significant downregulation of genes associated with activation of adaptive immunity after additional hemorrhage. The reduced expression of CD247 (CD3 subunit zeta) as part of the T-cell receptor-CD3 complex and of HLA class II histocompatibility antigens involved in MHC class II protein complex binding, such as H2-Aa, H2-Oa, H2-DMb2, and CD74, points to diminished antigen presentation. CD19 is upregulated during B-cell maturation, with a lower expression in immature than in mature B-cells; it has been shown to be especially critical for modulation of B-cell receptor-dependent and independent signaling [43]. The expression of CD22 is strictly limited to B-cells and negatively regulates B-cell receptor signaling [44]. The detected downregulation of genes involved in T- and B-cell activation is indicative of deficits in translation of antigen recognition into an effective cellular and humoral response and may provide an explanation of the impaired posttraumatic response of adaptive immunity [45].

Even though the present mouse model may be considered of clinical relevance, this study has some limitations. Analysis was restricted to one time point at four hours after trauma, and genomic expression was analyzed only in blood leukocytes. An implementation of a chronological sequence of analysis and a differentiation of immune cells could potentially provide a more detailed insight into the temporal changes of the genomic expression and specific alterations in cell types associated with the innate or adaptive immunity. In addition, the suitability of murine models for research on inflammation and immune responses remains controversial [46–49]. Furthermore, patient heterogeneity, including age, gender, medical history, and administration of blood transfusions as well as a pharmacopeia of drugs given to injured patients are normally not mimicked in experimental studies. Nevertheless, the high standardization of our model and the direct comparison between severely injured mice with and without hemorrhagic shock allowed us to specifically investigate the effects of an additional hemorrhage on the posttraumatic immune response.

## 5. Conclusion

Taken together, hemorrhage seems to aggravate the immune dysbalance in mice after severe injury, as molecular pathways and biological processes of the innate immune system were upregulated, whereas those of the adaptive immunity were downregulated already four hours after trauma by an additional hemorrhagic shock. Underlying mechanisms remain unknown and may include enhanced release of DAMPs or factors induced by tissue ischemia and reperfusion, making further investigations, potentially also in large animal models or patients essential. This dysregulation might play a contributing factor in the development of MODS in patients after trauma and hemorrhage, demonstrating the potential of not only diagnostic markers but also novel therapeutic targets in the innate and adaptive immunity.

## Data Availability

The microarray data used to support the findings of this study have been deposited in the Gene Expression Omnibus database (GEO accession number GSE160905). All other relevant data are included in this manuscript.
